# Activation of Waste Materials with Carbon(IV) Oxide as an Effective Method of Obtaining Biochars of Attractive Sorption Properties towards Liquid and Gas Pollutants

**DOI:** 10.3390/ma15228000

**Published:** 2022-11-12

**Authors:** Aleksandra Bazan-Wozniak, Judyta Cielecka-Piontek, Agnieszka Nosal-Wiercińska, Robert Pietrzak

**Affiliations:** 1 Faculty of Chemistry, Adam Mickiewicz University in Poznań, Uniwersytetu Poznańskiego 8, 61-614 Poznan, Poland; 2 Department of Pharmacognosy, Poznan University of Medical Sciences, Rokietnicka 3, 60-806 Poznan, Poland; 3 Faculty of Chemistry, Maria Curie-Sklodowska University in Lublin, Maria Curie-Sklodowska 3, 20-031 Lublin, Poland

**Keywords:** biochars, methylene blue, Langmuir model, thermodynamic study, NO_2_ adsorption

## Abstract

Biochars that are the subjects of this report have been obtained from the residue of supercritical extraction of common nettle seeds with CO_2_. The residue was subjected to direct activation with carbon(IV) oxide as an activator. The obtained biochars were found to have a specific surface area inthe range of 888–1024 m^2^/g and a basic surface. They were used for the adsorption of a liquid organic pollutant (methylene blue) and a gas inorganic pollutant (NO_2_). As follows from the test results, the biochars were able to adsorb 150–239 mg of the dye. The Langmuir model was found to better describe the adsorption experimental data, while the kinetics of the process was better described by the pseudo-second-order model. From the thermodynamic analysis, it was inferred that the adsorption of methylene blue from a water solution was an endothermic and spontaneous reaction. It was established that elevated temperature of activation and the presence of air stream during adsorption had a positive impact on the adsorption of NO_2_ by the biochars studied. The greatest sorption capacity of the biochars towards NO_2_ was 59.1 mg/g.

## 1. Introduction

The term “carbon adsorbents” refers to a large gamut of materials characterized by a high content of carbon, a large area and the ability toselectivelyadsorb molecules from liquid and gas phases [1,2]. Activated carbons are used for the removal of pollutants, separation of gas mixtures and recovery of substances [3,4,5]. Carbon adsorbents may also be used as adsorbents of metal ions [6] and compounds such as phenol [7] and bisphenol [8]. The continuously emerging new areas of potential use of porous materials of this type imply the need for the search for new carbon adsorbents dedicated to meeting particular demands [9,10]. The most often applied activation process is physical activation, which is a two-stage process comprising pyrolysis and activation [11]. This method permits obtaining activated carbons as pellets, grains or ash [12]. The main advantage of this method is the low cost of producing a unit of activated carbon, which has contributed to the large-scale use of this type of adsorbent in many branches of industry [13,14,15].

Unceasing interest in carbon adsorbents, including activated carbons, due to their numerous advantages and versatility, means that they continue to be a hot subject of research [16,17]. Much effort is directed to the improvement of their physicochemical properties and sorption capacity [18]. Another direction of research is optimization and modification of the methods of activated carbon production aimed at reducing production costs. One of the possibilities is reducing the physical activation process to a single stage. The raw product containing carbon in organic connections may be subjected to direct activation by the activating agent, e.g., carbon(IV) dioxide [19]. It would undoubtedly shorten the process of production of activated carbons and the use of waste products as precursors would additionally reduce the cost [20]. According to the authors of [21], the cost of production of 1 kg of carbonaceous adsorbent by chemical activation of sludge with the use of the conventional method was USD 70.67. It is reasonable to suppose that the cost of production of biochars by direct physical activation biomass will be much lower. The challenge is not only to find a suitable precursor of activated carbon but also to establish the right conditions forthe process [22]. As follows from a reviewof the literature in the field, biochars obtained from lignocellulosic biomass may be successfully used to remediate heavy metal contamination. Hoang et al. [23] have shown that adsorbents of this type show high selectivity towards heavy metal ions Cr, Pb, Cu, Cd, Hg and As. The authors of [24] have presented an interesting way of using biochars by proving that adsorbents of this type may increase the process of anaerobic digestion. The high porosity and basicity of the biochar surface are of key importance in this process.

The main purpose of this study was to prepare biochars by activatingthe residues of supercritical extraction of common nettle seeds with CO_2_. The obtained biochars were used for the removal of pollutants from air and a water solution. The pollutants used were a model water solution of methylene blue and NO_2_ from the air. The biochars were subjected to physicochemical studies in order to determine the elemental composition, textural features and number and types of oxygen functional groups in the structure.

## 2. Material and Method

### 2.1. Materials and Chemical Reagents

The biochar precursors were the residues of supercritical extraction of common nettle seeds with CO_2_ obtained from theNew Chemical Syntheses Institute of theŁukasiewicz Research Network. The starting material in a form of powder (0.10–0.95 mm) with amoisture content of 6.1 wt.% was washed with distilled water and dried at 105 °C for 48 h. Methylene blue, hydrochloric acid and sodium hydroxide were of analytical grade, purchased from Merck (Darmstadt, Germany). Deionized water was used in the preparation of the solutions.

### 2.2. Preparation of Biochars

**Figure 1:**
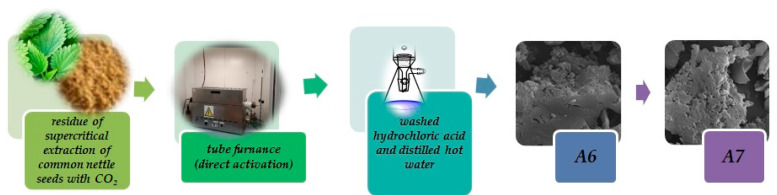
Scheme of biochar preparation.

The precursor was subjected to activation with CO_2_ in a tube reactor. The process was carried out at 600 (biochar A6) or 700 °C (biochar A7) for 45 min (10 °C/min). During the process, carbon(IV) oxide (250 mL/min) was blown through the tube furnace. Then,materials were cooled in the atmosphere of nitrogen and then removed from the furnace. The obtained biochars were washed with 0.200 L of 5% HCl and 20 L of distilled hot water (Figure 1).

### 2.3. Characterization of Precursor and Biochars

#### 2.3.1. Elemental Analysis

A Thermo Scientific FLASH 2000 Elemental Analyzer (OEA, Thermo Fisher Scientific, Waltham, MA, USA) was employed to establish the elemental composition of the precursor and biochars. The standard test method for ash was performed according to the ASTM D2866-94 Standard (2004).

#### 2.3.2. Low-Temperature Nitrogen Sorption

The porous structure of the obtained biochars wasdetermined by N_2_ adsorption/desorption isotherms. The measurements were performedat −196 °C on a QuantachromeAutosorb IQ apparatus (Boynton Beach, FL, USA). Prior to measurements, the biochar samples were degassed under vacuum for 6 h, at 300 °C. The calculations based on the BET method were used to quantify the surface areas of the biochars. The average pore size was evaluated from the adsorption branch of the isotherm by means of the BJH method.

#### 2.3.3. Scanning Electron Microscope (SEM)

SEM images were obtained using a scanning electron microscope (PHILIPS, Eindhoven, The Netherlands) in the following conditions: working distance of 14 mm, acceleration voltage of 15 kV and digital image recording by DISS.

#### 2.3.4. Acidic and Basic Character of the Precursor and Biochar Surfaces

The surface of oxygen functional groups was determined by the Boehm method [11].

### 2.4. Adsorption Studies

#### 2.4.1. NO_2_Adsorption

For evaluation of the biocarbon sorption capacity towards NO_2_, the samples were tested in dry and wet (70% humidity) conditions; more details are contained in our earlier work [11]. The concentration of gas NO_2_ was monitored using a Multi-Gas Monitor Q-RAE PLUS PGM-2000/2020 (RAE Systems, Sunnyvale, CA, USA). The tests were stopped at the breakthrough concentration of 20 ppm. The capacities of each biochar expressed as milligrams of NO_2_ per gram of the adsorbent were calculated according to the procedure described in [25].

#### 2.4.2. Methylene Blue (MB) Adsorption

The stock water solution of MB (1000 mg/L) was prepared, and from it, the solutions of concentrations inthe range 5–110 mg/L were made. In order to perform adsorption studies, portions of 20 mg of each biochar were mixed with 50 mL of methylene blue solution of the appropriate concentration. The samples were shaken for 1 day on a shaker (Heidolph, Schwabach, Germany, 200 rpm/min). After shaking, the solid contents were separated by centrifugation on a laboratory centrifuge (OHAUS, Parsippany, NJ, USA). The concentration of MB remaining in the solution was spectrophotometrically measured at λ_max_ = 665 nm on a Carry 100 Biospectrophotometer (Agilent, Santa Clara, CA, USA). The amount (q_e_) of MB adsorbed by 1 g of the biochar A6 or A7 was calculated using Formula (1):

(1) $$q_{e}=\frac{C_{0}-C_{e}}{M}\times V$$

where:

C_0_—initial concentration of MB solution (mg/L);
C_e_—MB concentration remaining in solution at equilibrium(mg/L);
M—mass of biochar(g);
V—volume of the solution (L).

Experimental data were fitted to the Langmuir (2) and Freundlich isotherms (3). In the Langmuir model, the adsorbed substance forms a single layer on the homogeneous adsorbate surface [26], while the Freundlich adsorption isotherm describes the process of adsorption on a heterogeneous surface [27].

(2) $$\frac{C_{e}}{q_{e}}=\frac{1}{K_{L}\times q_{\max}}+\frac{C_{e}}{q_{\max}}$$

(3) $${\mathrm{logq}}_{e}={\mathrm{logK}}_{F}+\frac{1}{n}{\mathrm{logC}}_{e}$$

where:

C_e_—concentrations of MB in the solution (mg/L);
q_e_—adsorption capacity (mg/g);
q_max_—MB monolayer adsorption capacity (mg/g);
K_L_—Langmuir adsorption constant (L/mg);
n_F_—Freundlich adsorption constant related to adsorption capacity;
K_F_—Freundlich adsorption constant related to sorption intensity (mg/g (mg/L)^1/n^).

We also used the non-linear Langmuir and Freundlich models.

To obtain the information on the mechanism of MB adsorption on the biochars obtained, two kinetic models were used: pseudo-first-order (PFO) and pseudo-second-order (PSO) [28]:

(4) $${\mathrm{logq}}_{e}={\mathrm{logK}}_{F}+\frac{1}{n}{\mathrm{logC}}_{e}$$

(5) $$\frac{t}{q_{t}}=\frac{1}{k_{2}q_{e}{}^{2}}+\frac{t}{q_{e}}$$

where:

q_t_—amount of MB adsorbed at time t (mg/g);
k_1_—pseudo-first-order model rate constant of adsorption (1/min);
k_2_—pseudo-second-order model rate constant of adsorption (g/mg×min).

The sorption studies of MB on the biocarbons obtained were also carried out at 25, 45 and 65 °C.

The thermodynamics of MB adsorption on the obtained biochars [29] was characterized by enthalpy (ΔH, kJ/mol), entropy (ΔS, J/K×mol) and Gibbs free energy (ΔG, kJ/mol) calculated from the following equations:

(6) $$\Delta G=-\mathrm{RT}\times {\mathrm{lnK}}_{C}$$

(7) $${\mathrm{lnK}}_{C}=\frac{\Delta S}{R}-\frac{\Delta H}{\mathrm{RT}}$$

where:

T—sorption temperature (K);
R—gas constant (83,144 J/K×mol);
K_C_—thermodynamic equilibrium constant expressed by q_e_/C_e_.

## 3. Results and Discussion

### 3.1. Characterization of the Precursor and Biochars

The elemental composition of the precursor and its acid–base properties are described in Table 1.

The content of mineral substance in the precursor was 3.2 wt.%, which means it was almost 3times lower than that in the residues of supercritical CO_2_ extraction of marigold [11]. The lower content of ash in the precursor used in this study was most probably a consequence of washing with hot distilled water. This procedure was not used for the precursor used in [11]. The precursor used was characterized by a low degree of carbonization, 49.4 wt.%, and a very low content of sulfur, 0.1 wt.%. The contents of other elements were as follows: H^daf^ = 9.9, N^daf^ = 4.6 and O^daf^ = 36.0 wt.%. As the content of acidic groups on the precursor’s surface was much higher (over 4-fold) than that of basic groups, its surface had a strongly acidic character.

**Figure 2:**
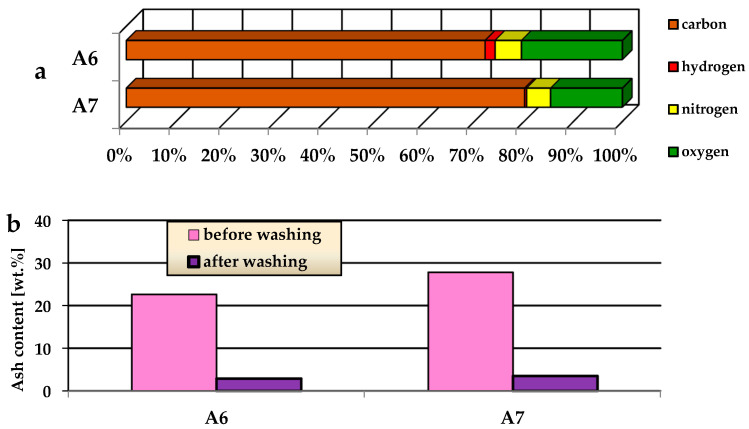
The elemental analysis (**a**) and ash content (**b**) of samples A6 and A7.

Elemental analysis of the biochars isdescribed in Figure 2a and shows the dominant presence of elemental carbon that varies in the range 72.1–79.8 wt.%. The activated carbons and biochars obtained as a result of one-stage activation (with CO_2_) or two-stage activation (pyrolysis and activation with CO_2_) contain about 80 wt.% of elemental carbon [30,31]. According to the data presented in Figure 2a, the increase in the activation temperature by 100 °C results in a significant decrease in the carbon content relative to that of sample A6, activated at 600 °C, which is interpreted as a consequence of enhanced aromatization of the carbon structure at higher temperatures [32]. As forthe effect of increased activation temperature on the content of nitrogen, it is slightly reduced (by 0.5 wt.%) in sample A7.

An important procedure in the preparation of the carbon adsorbents was their washing with a 5% solution of HCl and then with hot distilled water to remove a significant part of the mineral substance from the biochar structure (Figure 2b). As shown in Figure 2b, this procedure resulted in a considerable reduction in ash content, which was nearly 8 times smaller in the washed samples A6 and A7 relative to that in the samples not subjected to washing. For sample A6, the content of ash decreased from 22.6 to 2.9 wt.%, while for sample A7 the analogous decrease was from 27.8 to 3.5 wt.%. That is why the elemental analysis and the determination of the textural parameters, acid–base properties and adsorption capacity were performed for the samples washed with diluted hydrochloric acid and hot distilled water.

**Figure 3:**
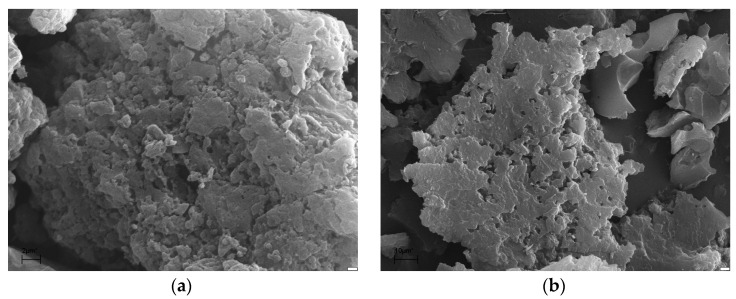
SEM micrographs of samples A6 (**a**) and A7 (**b**).

The porous structure of the biochars ispresented in Table 2. The SEM images are presented in Figure 3. The brighter fragments observed for biochars may be due to the presence of ash.

Upon activation of the precursor, the specific surface area, area of micropores and total pore volume of the biochars increase with increasing activation temperature. Sample A7 showed a greater specific surface area than biochar A6. An increase in the activation temperature by 100 °C results in the specific surface area increasingby almost 200 m^2^/g. The surface area of biochar A7 looks very good against a background of those of the other adsorbents described in the literature. S_BET_ of sample A7 is much larger than those of the carbon adsorbents obtained by activation of, e.g., hay [19] or pistachio nutshells [32], with CO_2_. Moreover, the S_BET_ of sample A7 is greater than those of the adsorbents obtained by much more expensive chemical activation [33,34]. For instance, Neme et al. [34] in their paper described the synthesis of an adsorbent from castor seed hulls by activation with H_3_PO_4_, using different amount ratios of the precursor to the activator. The greatest S_BET_ they obtained was 785 m^2^/g, which was determined in [33] for the activated carbon synthesized at the highest amount ratios heated at 700 °C for 60 min. The S_BET_ of the sample obtained from the castor seed hull was smaller than that of biochar A6 and sample A7. Moreover, we used a lower (600 °C) or the same (700 °C) temperature of activation and the process of physical activation, which is much cheaper than the chemical one. The specific surface area of our biochars is comparable to or greater than those of the commercial products [35,36] used for the removal of liquid and gas pollutants. The specific surface area of samples A6 and A7 was greater than that of the commercial carbon prepared from palm shell charcoal (838 m^2^/g) produced by physical activation [35]. In view of the above exemplary literature data, the method of activation of the residues of supercritical extraction of common nettle seeds with CO_2_ may be successfully used for the production of materials of comparable or even better textural properties than those described in the literature. As shown in Table 2, the mean diameters of the pores in the biochars we obtained are 4.1 nm and 3.5 nm for samples A6 and A7, respectively, which indicates the dominance of small mesopores.

**Figure 4:**
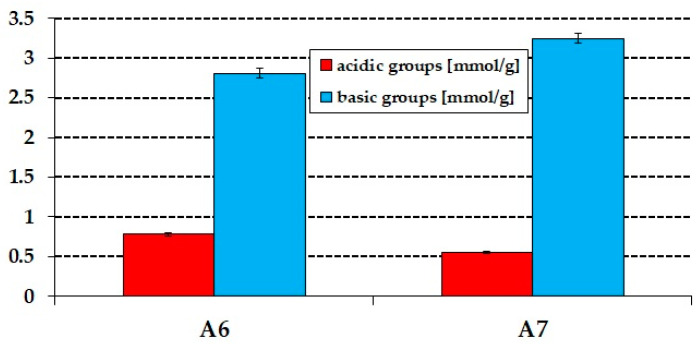
Acid–base properties of A6 and A7 samples obtained.

The chemical character of the surface of the obtained biochars provides information on the type of reactions taking place upon adsorption of pollutants. Of key importance is the content of oxygen functional groups that may be basic or acidic. The amount of basic and acidic oxygen functional groups was estimated by Boehm titration, theresults of which are presented in Figure 4. The activation of the precursor with CO_2_ was found to generate acidic and basic groups, but the sample activated atthe higher temperature exhibiteda decrease in the number of acidic groups and an increase in the number of basic ones. Sample A7 was also found to show richer chemistry of the surface; the total amount of oxygen functional groups was 3.80 mmol/g, including 0.55 mol/g of acidic ones and 3.25 mmol/g of basic ones. Sample A6 contained 0.78 mmol/g of basic groups and 2.81 mmol/g of basic ones.

### 3.2. NO_2_Adsorption

The obtained biochars were tested as adsorbents of the gas pollutant NO_2_. At present, it is important to have NO_2_ adsorbents ready to use as in a time of economic crisis, people may resort to using all kinds of products for heat generation. Adsorption of NO_2_ by samples A6 and A7 was tested in wet and dry conditions, and the results are presented in Table 3.

The effectiveness of NO_2_ removal was observed to depend on the activation temperature and conditions of adsorption. Irrespective of the conditions of the process, the sorption capacity of sample A6 was lower than that of A7. In the process with no access toair, the sorption capacity of A7 increased by 7.4 mg, while in wet conditions it increased by 16.3 mg. The greater sorption capacity of A7 is a consequence of its better-developed porous structure; moreover, biochar A7 has more surface oxygen functional groups that may interact with the pollutant [37]. The sorption capacities of adsorbents A6 and A7 (irrespective of the conditions of adsorption) were greater than those of the activated carbons obtained in our earlier studies [32]. Moreover, the samples prepared by activated carbons obtained by direct activation of pistachio nutshells [32] needed a higher activation temperature and longer time of heating than the corresponding values used in this study for the residues of supercritical CO_2_ extraction of common nettle seeds. Therefore, the activation method proposed may be considered effective.

The effectiveness of NO_2_ removal of the biochars obtained wasdependent on the conditions of adsorption. The sorption capacities of samples A6 and A7 were over twice greater if the process was performed in the presence of steam. For example, a doubling in the sorption capacity towards NO_2_ was not noted for carbonaceous adsorbents obtained by direct activation of hay (microwave method) [19] and adsorbents prepared by direct activation from pistachio nutshells (conventional method) [32]. Biochar A6 adsorbed 20.1 mg of NO_2_ in dry conditions and 42.8 mg NO_2_ in the presence of steam. The analogous values for sample A7 were 28.7 mg/g and 59.1 mg/g in dry and wet conditions, respectively. Analysis of the results suggests that sample A6 proved less susceptible to the impact of the conditions of NO_2_adsorption than A7.

**Figure 5:**
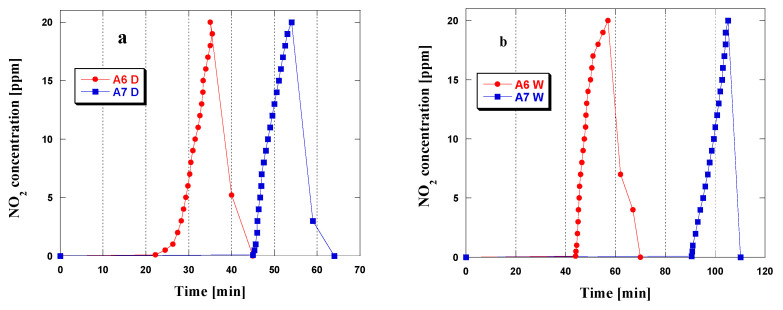

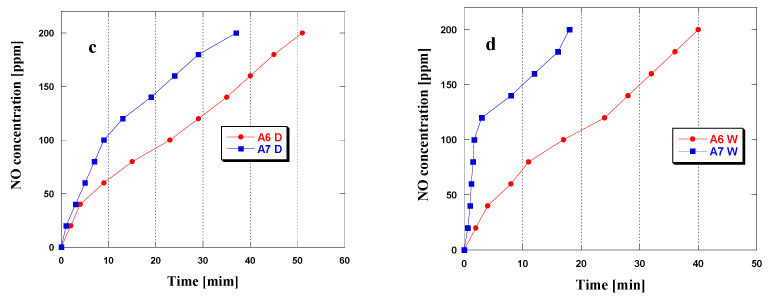
NO_2_ and NO breakthrough curves obtained during adsorption in dry (**a**,**c**) and wet (**b**,**d**) conditions for the biochars obtained.

The isotherms of NO_2_ adsorption/desorption are presented in Figure 5a,b. Irrespective of the conditions of the process, their shapes are similar. The ideal adsorbent should be characterized by the breakthrough curve with zero concentration of NO_2_ for a long time, followed by the rapid breakthrough of the carbon bed and a fast increase in the concentration of the gas studied [38]. In wet conditions (Figure 5b), the shape of the isotherms is close to such an ideal shape. The curve obtained for sample A7 is particularly close to the ideal shape: the concentration of gas was 0 ppm for nearly 90 min and then it rapidly increased to 20 ppm. The character of NO_2_ curves recorded for sample A7 in wet conditions when compared to that obtained for bio-activated carbon presented in [19] confirms the effectiveness of the applied method of activation of the starting material. For sample A7, the 0 ppm concentration of gas was maintained for much longer than that for the sample obtained from hay, and the time of adsorption was shorter. The time of zero concentration of NO_2_ was longer for the adsorption carried out in the presence of steam, which consequently gave greater sorption capacities in wet conditions (Table 3). On the basis of the shapes of the adsorption/desorption isotherms, it can be concluded that both in dry and wet conditions, after the NO_2_ influx to the carbon bed is cut off, the concentration of NO_2_decreasesto zero. This may indicate that NO_2_ was strongly bound in the structure of samples A6 and A7 or that it was chemisorbed [37]. It should be emphasized that the shape of NO2 adsorption/desorption curves recorded for samples A6 and A7 implies that the mechanism of NO_2_ adsorption on their surfaces is the same.

Moreover, it can be inferred that the following reactions take placein dry conditions [39]:

(8) $$C\;+2{\mathrm{NO}}_{2}\to {\mathrm{CO}}_{2}+2\mathrm{NO}$$

(9) $$C\;+{\mathrm{NO}}_{2}\to \mathrm{CO}+\mathrm{NO}$$

In the presence of steam, the adsorption of NO_2_ may lead to the generation of a mixture of acids:

(10) $$3{\mathrm{NO}}_{2}+H_{2}O\to 2{\mathrm{HNO}}_{3}+\mathrm{NO}$$

(11) $$2{\mathrm{NO}}_{2}+H_{2}O\to {\mathrm{HNO}}_{3}+{\mathrm{HNO}}_{2}$$

which would lead to greater sorption capacities in wet conditions [39,40].

In the process of NO_2_ adsorption, changes in the NO concentration were also observed in dry (Figure 5c) and wet (Figure 5d) conditions. The shapes of the curves recorded imply that the biochars show a rather good ability to reduce NO_2_ to NO, but for sample A7, this ability is stronger, irrespective of the conditions of adsorption. Additionally, in the process run in wet conditions, the process of reduction is more effective, which is well pronounced for sample A7, as the concentration of 200 ppm (NO) in the process of adsorption on this sample in wet conditions was achieved in the time twice shorter than that in dry ones.

Table 4 presents the sorption capacities of different selected adsorbents towards NO_2_. Biochar A7 is less effective in the removal of NO_2_ than the adsorbents prepared in our earlier studies [11,25]. The NO_2_ adsorption by the biocarbon obtained from marigold [11] was performed in mixed dry conditions; that is, prior to adsorption, the biocarbon surface was wetted with air of 70% humidity for 30 min. This procedure undoubtedly had a great impact on the sorption capacity of this adsorbent, which was twice greater than that of sample A7. The activated carbon prepared from hops [25] was subjected to chemical activation of the precursor by Na_2_CO_3_. The synthesis of this activated carbon was performed using the activator to precursor amount ratio of 3:1. The sorption capacity of the activated carbon described in [25] towards NO_2_ was 155.3 mg/g. However, the synthesis of this adsorbent was much more time-consuming and expensive than the synthesis of biochars presented in this paper. The sorption capacity of the activated carbon obtained from sawdust pellets (54.7 mg/g) [41] was similar to that of biochar A7 (59.1 mg/g). Much lower effectiveness in the removal of NO_2_was shown bythe adsorbent obtained by chemical activation of waste tires with KOH [42]; it was over 5times lower than that of sample A7.

### 3.3. Methylene BlueAdsorption

**Figure 6:**
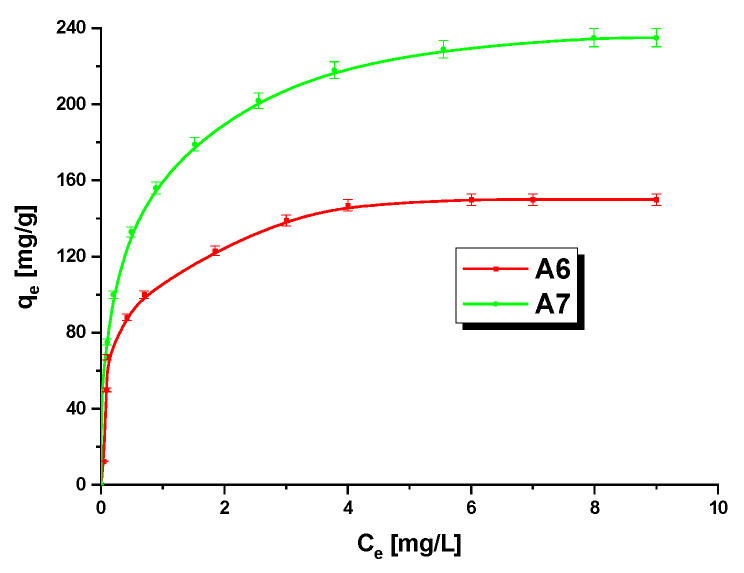
Isothermsfor adsorption of methylene blue on A6 and A7 samples.

The effect of the initial concentration of MB on the sorption capacities of the samples studied was checked (MB concentrations were 5–110 mg/L). According to the character of the curves presented in Figure 6, the efficiency of MB removal decreased with theincrease in theinitial MB concentration. Thisis a consequence of a decrease in the number of active centers available during the adsorption process. The higher effectiveness of MB removal at its low initial concentrations follows from a smaller ratio of the number of MB molecules to that of the active centers on the biochar surface [43]. The temperature of activation also had a considerable impact on the sorption capacities of the biochar samples. Sample A6 was able to adsorb 150 mg of MB, while sample A7 (activated at 700°C) showed asorption capacity of 239 mg/g. Therefore, in addition to its greater effectiveness in NO_2_ adsorption,biochar A7 proved to be a more effective adsorbent towards methylene blue in water solution.

The experimental data describing the adsorption process were fitted by the Langmuir and Freundlich models (Table 5).

**Figure 7:**
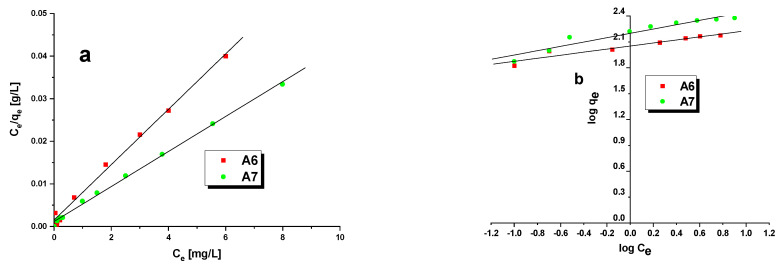
Linear fitting of isotherm models: Langmuir isotherm (**a**) and Freundlich isotherm (**b**).

The Langmuir equation assumes that the adsorption takes place in a monolayer with homogeneously distributed active centers forming on the adsorbent surface and that the heat of adsorption does not depend on the area of the adsorbent covered. The maximum sorption capacity q_max_ and the value of constant K_L_ describing affinity between MB and the biochar can be read from the slope of the dependence plotted in Figure 7a and the intercept of this plot with the y-axis. The Freundlich equation assumes that the adsorption takes place in a multilayer structure in which the adsorbate is adsorbed in a heterogeneous system [43]. The strength of adsorption (parameter n) and sorption capacity (parameter K_F_) are read from the plot presented in Figure 7b. According to the R^2^ values, included in Table 5, the correlation of the experimental data to the Langmuir model predictions is stronger: for sample A6, the value of R^2^ is 0.9950, while for A7, it is 0.9967. For comparison, the R^2^ values obtained assuming the Freundlich model for samples A6 and A7 are 0.9215 and 0.9240, respectively. Thus,the MB adsorption on the biochars studied is the process of monolayer chemical adsorption. The process of adsorption on sample A7 was better fitted by the Langmuir model than that on sample A6. Moreover, a review of the literature shows that the adsorption of MB on porous adsorbents is much more often described by the Langmuir model [17,19,25,44]. The values of the maximum sorption capacity at equilibrium calculated assuming the Langmuir model (A6–153.85 mg/g, A7–243.90 mg/g) are close to the experimental values (Figure 6). As the value of the K_L_ constant was higher for sample A6, the bonds between MB and the biochar surface arestronger for this sample. The last parameter calculated assuming the Langmuir model was the dimensionless coefficient R_L_, whose values were in the range from 0 to 1,indicating favorable adsorption. The value of 1/n, characterizing the strength of adsorption, was in the range 0 < n < 1, indicating that the chemical bonds formed between the biochars and MB are strong [45]. Taking into account the value of K_F_, it can be inferred that biochar A7 was more selective towards MB than sample A6.

**Figure 8:**
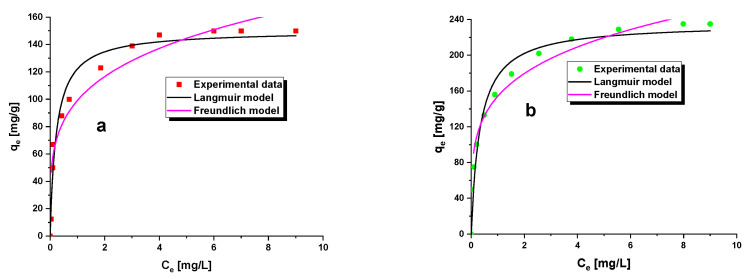
Non-linear fitting of isotherm models: Langmuir isotherm (**a**) and Freundlich isotherm (**b**).

Moreover, we determined the parameters of the nonlinear forms of the Langmuir and Freundlich equations [46]. The results are presented in Table 6 and Figure 8. As follows from these results, the Langmuir model better describes the experimental data than the Freundlich model, although the correlation coefficient (R^2^) values are much smaller than those obtained for the linear form of the Langmuir equation. As follows from a comparison of the fit of results obtained for both biochars to the nonlinear Langmuir equation, the fit was better for sample A6, as indicated by a higher value of R^2^calculated for the results of this sample.

**Figure 9:**
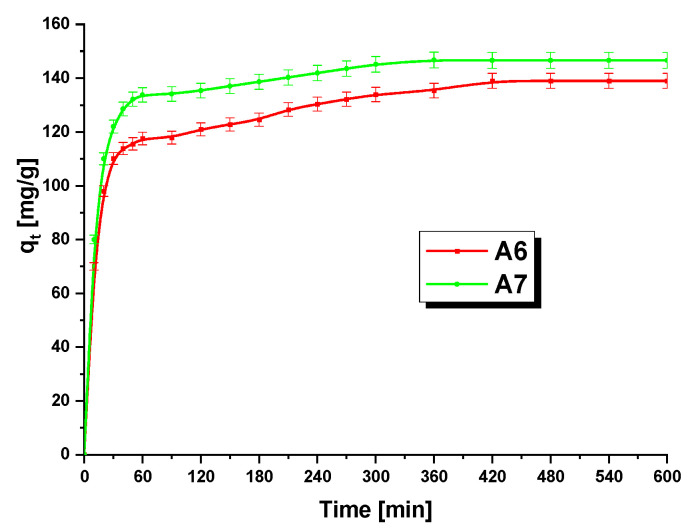
Influence of contact time on the adsorption of methylene blue on samples A6 andA7 (biochar mass: 20 mg, initial dye solution concentration: 60 mg/L, volume of methylene blue solution: 50 mL, temperature: 22 ± 1 °C).

In the next stage, the impact of contact time on the adsorption of methylene blue on A6 and A7 samples was evaluated. The experimental data are shown in Figure 9. The course of the isotherms recorded for samplesA6 and A7 implies that in the first 60 min of the process, the sorption capacity of the biochars towards MB was rapidly increasing, which is related to a large number of free active centers. After this time, the effectiveness of the process started decreasing, and after 7 h, a state of adsorption equilibrium was reached. This decrease is a consequence of the fact that MB molecules gradually occupy the active centers on the biochar surface, slowing down the process of adsorption until the equilibrium is reached [29]. At the stage of adsorption equilibrium, a higher sorption capacity was shown bysample A7. A saturation point of 480 min has also been noted for the activated carbons described by AlOthman et al. [47]. However, the samples they studied showed lower sorption capacities towards methylene blue at the state of equilibrium (varied from 76 to 128.89 mg/g) than those obtained for samples A6 and A7.

**Figure 10:**
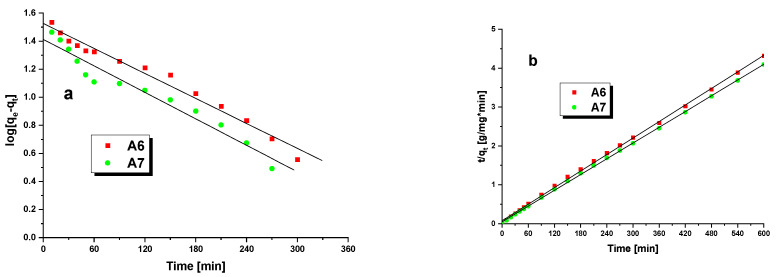
Pseudo-first-order (**a**) and pseudo-second-order (**b**) kinetic plots for adsorption of methylene blue on samples A6 and A7.

Knowing the impact of the time of adsorbent/adsorbate contact on the adsorbent sorption capacity, we were able to calculate the kinetic parameters for the two models of kinetics. The calculated values are given in Table 7, while the plots illustrating the fits with these two models are presented in Figure 10.

According to the above-presented results, the values of q_e,exp_for samples A6 and A7 are significantly different from q_e,cal_ predicted by the PFO model. In addition, the value of R^2^ for this model is much lower than 0.999, which excludes the fit to experimental data. The sorption capacity predicted by the PSO model is much closer to the experimental results, which is also confirmed by the value of R^2^. Therefore, it may be inferred that the adsorption of MB from water solution has the character of chemisorption [48]. Data presented in Table 7 show that biochar A7 shows a higher affinity to the PSO model, as the value of R^2^for this sample is higher. However, a smaller difference between q_e,exp_ and q_e,cal_ was noted for sample A6.

**Figure 11:**
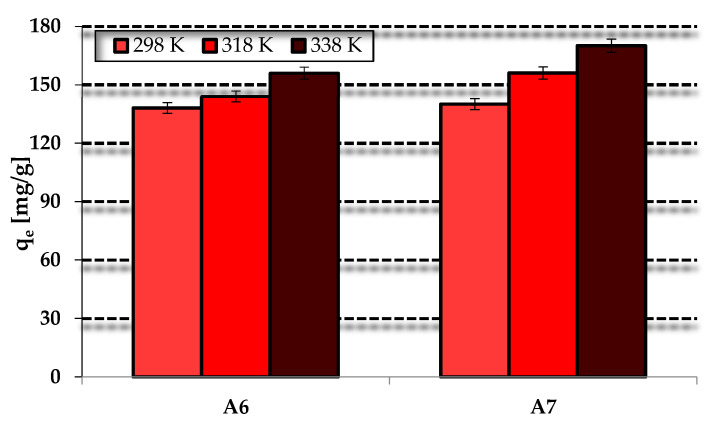
Effect of temperature on the adsorption of methylene blue on A6 andA7 samples (biochar mass: 20 mg, initial dye solution concentration: 60 mg/L, volume of methylene blue solution: 50 mL).

Next, we checked the effect of the temperature of adsorption on the effectiveness of removal of MB from its water solution by the biochars obtained. Three temperature variants were followed (Figure 11).

According to the experimental data presented in this figure, the temperature of adsorption has no significant impact on the effectiveness of MB adsorption on the biochars studied. However, with anincrease in thetemperature of adsorption, the sorption capacities of the biochars A7 and A6 increase; the increase was greater for A7. It should be noted that A6 and A7 increase their sorption capacity with increasing temperature of adsorption, while for the biochars described in [49], the opposite tendency was observed; their sorption capacities decreased with increasing temperature of adsorption.

The measurements also permitted the determination of thermodynamic parameters: Gibbs free energy, enthalpy and entropy (Table 8) [50]. As the values of ΔG vary in the range of −20 to 0 kJ/mol, it was concluded that the process has a physical character. With increasing temperature, ΔG takes more negative values, which means that the process becomes increasingly spontaneous, and it is more spontaneous for sample A7. Positive values of ΔH confirm that the adsorption of MB from water solution is endothermic and needs energy input [50]. A comparison of the ΔH values obtained for the two biochars shows that a greater energy input is needed for adsorption on sample A7.

The sorption capacity of biochar A7 towards methylene blue was compared with the results obtained for other materials (Table 9). The effectiveness of methylene blue removal by adsorption on sample A7 was much lower than that of the biochar obtained in a one-step template method [51] using heavy bio-oil produced from biomass pyrolysis as a precursor [51]. Zang et al. have proved that this material was able to adsorb 411 mg/g of methylene blue. Similar to that for biochar A7, the adsorption of methylene blue on this material was described by the Langmuir and pseudo-second-order models. A similar value of sorption capacity to that of sample A7 was reported for the activated carbon obtained by chemical activation of *Dipterocarpus alatus* fruit with ZnCl_2_ [52]. However, it should be noted that the synthesis of the latter adsorbent is more expensive than that of the synthesis of our samples, A7 and A6. Biochar A7 showed over twice greater effectiveness in the removal of methylene blue from its water solution than the coal gangue-based zeolite granules (108 mg/g) [53]. The adsorbent described in [54] showed a much lower sorption capacity than that of sample A7. Soury et al. [54] have reported that the adsorption of methylene blue on this material was described by the Freundlich isotherm.

## 4. Conclusions

The paper presents the obtaining of biochars by activation with CO_2_ of the residue of supercritical CO_2_ extraction of common nettle seeds and characterization of their performance as adsorbents of methylene blue and NO_2_. The effect of the activation temperature on textural parameters and sorption properties of the biochars prepared was studied.

Higher sorption capacities towards the pollutants studied were shown bysample A7. However, the two biochars studied showed well-developed surface area with the dominant presence of small mesopores. Their surface was found to have basic character. The biochars were tested as adsorbents of methylene blue and NO_2_. The effectiveness of NO_2_ removal was found to depend on the activation temperature and (dry or wet) conditions of adsorption. The increase in the activation temperature was favorable for effectiveness of NO_2_ removal from the air by the biochars obtained; moreover, the presence of steam in the process of adsorption had a favorable impact on the sorption capacities of the biochars. The process of adsorption of methylene blue on the biochars studied can be described by the Langmuir isotherm and pseudo-second-order kinetic model. According to the determined thermodynamical parameters, the process of the adsorptionof methylene blue from water solution is endothermic and spontaneous.

## Figures and Tables

**Table 1:** Characterization of the precursor.

Sample	Ash	C^daf,1^	H^daf^	N^daf^	S^daf^	O^daf,2^	Acidic Groups (mmol/g)	Basic Groups (mmol/g)
precursor	3.2	49.4	9.9	4.6	0.1	36.0	2.01	0.48

^1^dry ash-free basic, ^2^ by difference.

**Table 2:** Textural parameters of samples A6 and A7.

Biochar	Surface Area ^1^ (m^2^/g)	Total Pore Volume (cm^3^/g)	Average Pore Diameter (nm)	Micropore Area (m^2^/g)
**A6**	888	0.55	4.1	689
**A7**	1024	0.73	3.5	903

^1^ error range between 2 and 5%.

**Table 3:** NO_2_ breakthrough capacities of A6 and A7 samples obtained.

Biochar	Dry Conditions [mg/g]	Wet Conditions [mg/g]
A6	20.1	42.8
A7	28.7	59.1

**Table 4:** NO_2_ sorption capacity of adsorbents.

Material/Sample	Adsorption Capacity (mg/g)	References
marigold	102.1	[11]
hops	155.3	[25]
zirconium—carboxylic ligand	154	[40]
sawdust pellets	54.7	[41]
waste tires	11.4	[42]
A7	59.1	(This study)

**Table 5:** Parameters of linear form Langmuir’s and Freundlich’s equations.

Isotherms	Parameters	A6	A7
*Langmuir*	R^2^	0.9950	0.9967
	q_m_(mg/g)	153.85	243.90
	K_L_(L/mg)	0.0281	0.0168
	R_L_	0.3086–0.8772	0.3511–0.7485
*Freundlich*	R^2^	0.9215	0.9240
	K_F_(mg/g(L/mg)^1/n^)	112.90	158.45
	1/n	0.1771	0.2512

**Table 6:** Parameters of non-linear form Langmuir’s and Freundlich’s equations.

Isotherms	Parameters	A6	A7
*Langmuir*	R^2^	0.9677	0.9631
	q_m_(mg/g)	152.07	240.73
	K_L_(L/mg)	4.3612	2.9817
*Freundlich*	R^2^	0.9117	0.9458
	K_F_(mg/g(L/mg)^1/n^)	99.01	154.12
	1/n	0.2351	0.2204

**Table 7:** Kinetic parameters for the adsorption of methylene blue.

Isotherms	Parameters	A6	A7
	q_e,exp_(mg/g)	138.99	146.60
*Pseudo-first-order*	R^2^	0.9769	0.9358
	k_1_(1/min)	0.0059	0.0083
	q_e,cal_(mg/g)	35.61	27.69
*Pseudo-second-order*	R^2^	0.9993	0.9998
	k_2_(g/mg×min)	0.0007	0.0009
	q_e,cal_(mg/g)	140.85	149.25

**Table 8:** Thermodynamic parameters of the adsorption of methylene blue on A6 andA7 samples.

Sample	Temperature (K)	ΔG (kJ/mol)	ΔH (kJ/mol)	ΔS (J/mol × K)
**A6**	298	−8.77	16.99	86.13
	318	−10.23		
	338	−12.24		
**A7**	298	−11.59	21.91	111.91
	318	−13.35		
	338	−16.11		

**Table 9:** Methylene blue sorption capacity of selected adsorbents.

Material/Sample	Adsorption Capacity (mg/g)	References
heavy bio-oil	411	[51]
*Dipterocarpus alatus* fruit	269	[52]
coal gangue-based zeolite granules	108	[53]
*meso*-tetrakis(2,4,6-trimethylphenyl) porphyrinto) zinc(II) supported sodium alginate gel beads	52	[54]
A7	239	(This study)

## Data Availability

Data arecontained within the article.
